# The reallocation effect of China's provincial power transmission and trade on regional heavy metal emissions

**DOI:** 10.1016/j.isci.2021.102529

**Published:** 2021-05-11

**Authors:** Wendong Wei, Zengcheng Xin, Yong Geng, Jiashuo Li, Mingtao Yao, Yaqin Guo, Pengfei Zhang

**Affiliations:** 1 School of International and Public Affairs, Shanghai Jiao Tong University, Shanghai 200030, China; 2 SJTU-UNIDO Joint Institute of Inclusive and Sustainable Industrial Development, Shanghai Jiao Tong University, Shanghai 200030, China; 3 China Institute for Urban Governance, Shanghai Jiao Tong University, Shanghai 200030, China; 4 Business School, University of Shanghai for Science and Technology, Shanghai 200093, China; 5 Institute of Blue and Green Development, Shandong University, Weihai 264209, China; 6 School of Business, Shandong University, Weihai 264209, PR China; 7 Academy of Macroeconomic Research, National Development and Reform Commission, Beijing 100038, China; 8 School of Energy and Power Engineering, Huazhong University of Science and Technology, Wuhan 430074, China

## Abstract

Coal-fired power plants (CFPPs) are key point sources to atmospheric heavy metal (HM) emissions in China. Unevenly distributed CFPPs lead to large-scale interregional power transmission, as well as corresponding environmental emissions transfer. However, the effect of power transmission on HM reallocation remains poorly understood. Here, we traced HM (including Hg, As, Se, Pb, Cd, and Cr) emission flows through electricity transmission and regional trade and calculated China's multi-perspective electricity-related HM emissions from 2010 to 2015. Results show that in 2015, power transmission and regional trade caused 226.5 t (14% of total emissions) and 453.6 t (28%) of HM emission flows, respectively, leading to great differences in provincial HM emissions under different perspectives (e.g., Beijing's consumption-based emission was 15.5 times higher than the city's production-based emission in 2015). Our study provides valuable insights for fairly allocating provincial HM emission reduction responsibility and formulating synergistic emission mitigation strategies among regions.

## Highlights

• Declining HM emission factors greatly offset China's HM emission growth.
• Regional HM emissions under different perspectives varied substantially.
• Power transmission and regional trade caused large-scale HM emission flows.
• Developed provinces transferred HM emissions to less developed provinces.

## Graphical abstract

**Figure:**
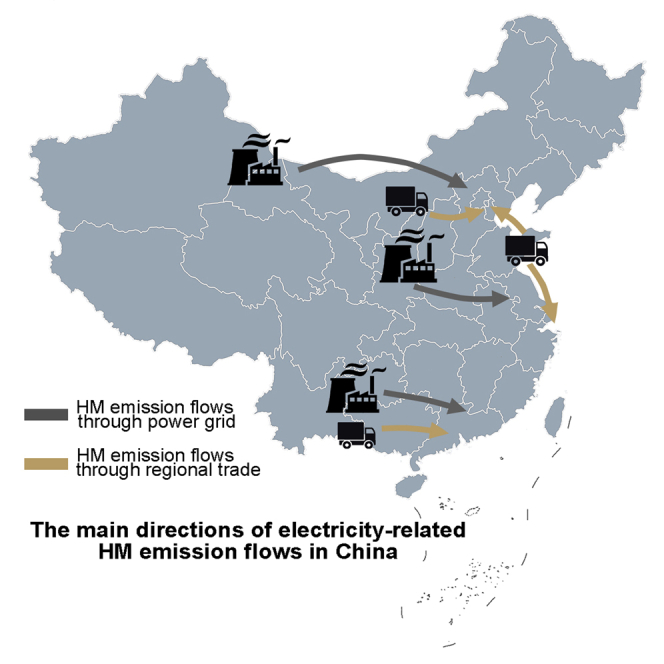

## Introduction

China has the largest number of coal-fired power plants (CFPPs), which are responsible for more than half of the country's coal consumption (Peng et al., 2020). Meanwhile, these CFPPs are a major source of various air pollutants, including heavy metal (HM) emissions, sulfur dioxide, particular matters and nitrogen oxides, which pose serious threats to human health (Chen et al., 2019; Feng et al., 2013; Jiang et al., 2020; Zhu et al., 2016). Although the Chinese government has made great achievements in the green transformation of its power sector (Li et al., 2020; The State Council, 2013; Wei et al., 2021), it is still challenging to control the increasing HM emissions from CFPPs (Li et al., 2019; Tian et al., 2011).

Accurate accounting of regional HM emissions is the basis for allocating environmental responsibilities and setting emission mitigation targets (Qian et al., 2021). Many studies have investigated HM emissions from power generation. For instance, Liu et al. (2018a) and Li et al., 2020 calculated the Hg emission generated from China's power plants. Zhou et al. (2019) calculated six HM emissions (namely, Hg, As, Pb, Cr, Se, and Cd) generated by decommissioned CFPPs in China in 2010 and evaluated the impacts of shutting down these CFPPs on HM emissions. However, these studies only focus on production-based (end-of-pipe) electricity-related HM emissions (PE-HM emissions) and ignore the reallocation effects of power transmission and regional trade on regional HM emissions (Ma and Zhang, 2019), which may lead to cross-regional HM emission leakage (similar to carbon leakage) (Li et al., 2013; Naegele and Zaklan, 2019). On the one hand, by purchasing electricity from other regions, HM emissions from local power generation plants can be prevented. On the other hand, by providing less electricity-intensive goods (such as software) to other regions and purchasing electricity-intensive goods (such as aluminum products) through regional trade, one region can reduce its local electricity demand, as well as its electricity production and associated HM emissions (Wei et al., 2020a). Ignoring the HM emission flows through power transmission and regional trade may lead to an unfair allocation of regional environmental responsibilities associated with HM emissions, resulting in regional environmental inequality. It can also undermine the effectiveness of HM emission reduction policies (Wei et al., 2020b; Zhang et al., 2020).

This study aims to quantify China's HM emission flows through power transmission and regional trade and establish a multi-perspective HM emission inventory (including the production side, supply side, and consumption side), which can help reveal both temporal and spatial evolution characteristics of HM emission flows in China. First, we calculate China's provincial PE-HM emissions for the period of 2010–2015 using the dynamic technology-based emission factor model (Zhang et al., 2015) (Hg, As, Se, Pb, Cd, and Cr are considered in this study). Then, we use the logarithmic mean Divisia index (LMDI) method to quantify the contributions of driving factors to PE-HM emissions changes, in which HM emission factors of coal, coal-fired power generation efficiency, power generation structure, and total power generation are considered. In addition, the network approach developed by Qu et al. (2017) is applied to trace the interprovincial HM emission flows through interconnected power grids and account the HM emissions induced by regional electricity consumption (SE-HM emissions). Finally, the environmentally extended multi-regional input-output (MRIO) model is adopted to trace the HM emission flows embodied in interprovincial trade and uncover China's provincial consumption-based electricity-related HM emissions (CE-HM emissions). Several HM emission mitigation suggestions are then proposed from different perspectives, which could promote more reasonable and effective policies for mitigating regional electricity-related HM emissions.

## Results

### China's provincial PE-HM emissions

China's power generation from CFPPs increased by 23.8% from 3416.5 TWh in 2010 to 4230.6 TWh in 2015, and the corresponding emissions of PE-HM decreased by 9.4% from 1759.5 t in 2010 to 1594.0 t in 2015. In 2010, PE-Hg, PE-As, PE-Se, PE-Pb, PE-Cd, and PE-Cr emissions reached 108.5, 278.7, 435.5, 537.3, 9.1, and 390.5 t, respectively; in 2012, these 6 PE-HM emissions increased by 6.5%, 8.6%, 7.1%, 8.6%, 7.9% and 4.8% (based on data for 2010), respectively; in 2015, levels of these 6 PE-HM emissions decreased by 17.0%, 15.0%, 13.7%, 14.1%, 14.5% and 19.8% (based on data for 2012), respectively. Detailed HM emissions data are listed in the Tables S1–S3.

**Figure 1:**
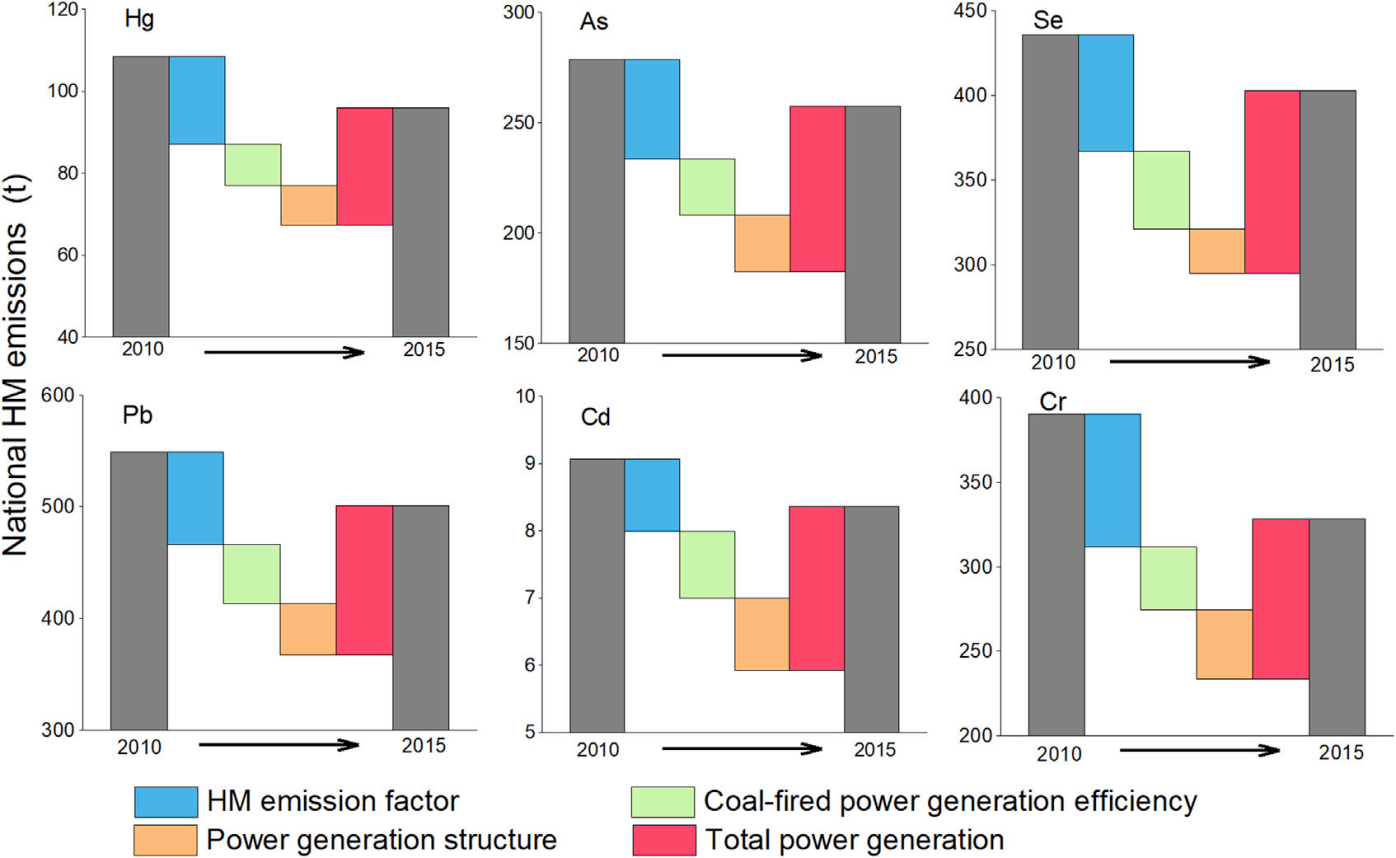
Contributions of driving factors of HM emissions from China's CFPPs.

We find that the decreased HM emission factors, the improvement of coal-fired power generation efficiency and the improvement of power generation structure (decreased proportion of coal-fired power to the total power generation) contributed to the reduction of PE-HM emissions, among which the reduction of HM emission factors contributed the most (see Figure 1). On the other hand, the growth of total power generation led to an increase of the total PE-HM emissions. For example, total power generation growth contributed 28.5 t of the PE-Hg emissions increase from 2010 to 2015, while HM emission factors (−21.5 t), coal-fired power generation efficiency (−10.0 t) and power generation structures (−9.6 t) offset the growth of PE-Hg emissions. Inner Mongolia, Heilongjiang and Zhejiang made great progress in reducing their EFs of coal-related HM emissions. For example, EFs contributed to the reduction of 2.7 t PE-Hg emissions in Inner Mongolia and 18.4 t PE-Pb emissions in Heilongjiang. Total power generation had a positive impact on regional PE-HM emissions in all the regions except Shanghai. Inner Mongolia and Shandong experienced the largest growth in total power generation (power generation in these two regions increased by more than 130 TWh during 2010–2015). However, power generation efficiency and structure had little influence on PE-HM emissions.

Shanxi, Inner Mongolia, Jiangsu, Zhejiang, Anhui, Shandong, Henan, and Guangdong were the main sources of PE-HM emissions. For each type of HM emission, these 8 provinces contributed more than half of the total emission, mainly due to significant coal consumption for power generation in these provinces (Tian et al., 2015), which collectively contributed 56% of coal consumption from all the China's CFPPs in 2015. Different kinds of HM emissions show different characteristics: from 2010 to 2015, Inner Mongolia was the province with the highest emissions of PE-Hg, PE-As and PE-Pb; Anhui was the province with the highest emission of PE-Se; Shanxi was the province with the highest PE-Cd emission; Zhejiang and Jiangsu were the provinces with the highest PE-Cr emission in 2010 and 2015, respectively.

We also calculated the HM emissions per unit of electricity at the provincial level and found that during 2010–2015, Anhui was the province with the highest PE-Hg and PE-Se intensity, Jilin was the province with the highest PE-As emission intensity, and Ningxia was the province with the highest PE-Cd emission intensity.

### HM emission flows through electricity transmission

The scale of interprovincial electricity transmission in China increased from 587.9 TWh in 2010 to 948.2 TWh in 2015 (China Electricity Council, 2011-2016a). China's net HM emission flows caused by electricity transmission reached 218 t (including 15.2 t Hg, 37.4 t As, 50.3 t Se, 67.6 t Pb, 1.0 t Cd and 46.5 t Cr) in 2010. This number reached 247 t in 2012 but dropped to 217 t in 2015.

**Figure 2:**
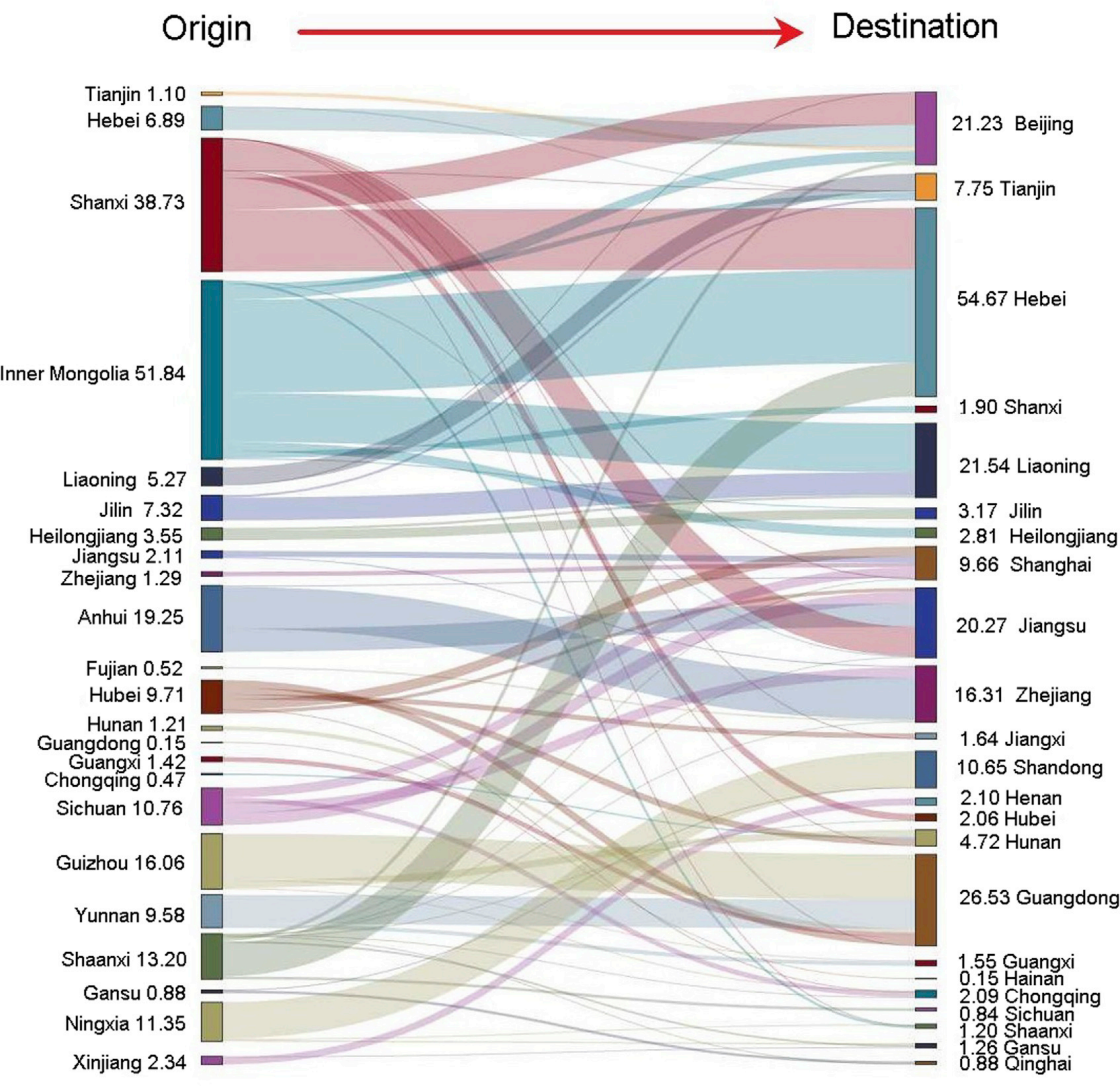
Net HM emission flows through interprovincial power transmission in 2015 (Unit: t; those emission flows of less than 0.1 t are not shown; those emission flows in 2010 and 2012 are illustrated in Figures S1 and S2).

From 2010 to 2015, the general direction of electricity transmission in China was oriented from the less developed central and western provinces to the more developed eastern provinces (see Figure 2), especially from provinces with rich coal resource (such as Inner Mongolia and Shanxi) to Beijing, Tianjin, Hebei, Liaoning, Shandong, and other provinces and from Yunnan and Guizhou to Guangdong. The direction of HM emission flows through electricity transmission presented similar patterns. In addition, several southern provinces such as Anhui, Guizhou and Yunnan were also the main provinces with net outflows of HM emissions.

We found that Inner Mongolia, Shanxi and Anhui were the provinces with the largest net HM emission outflows from 2010 to 2015, and the total net outflow of these three provinces accounted for more than 50% of the domestic net outflow. The main provinces with net HM emission inflows were Hebei, Guangdong, Liaoning, Beijing and Jiangsu, and the net inflow proportion of these five provinces to the annual domestic net inflow increased from 57.1% in 2010 to 66.7% in 2015.

Moreover, the main provinces with net HM emissions inflows and outflows in each year were relatively fixed, and the net HM emission outflows from Inner Mongolia, Shanxi and Anhui were relatively stable. However, the main provinces with net HM emission inflows (i.e., Hebei, Guangdong, Liaoning, Beijing and Jiangsu) had increasing proportions of net inflows. It is worth noting that compared to those of other provinces, the net HM emission inflow in Hebei increased rapidly from 11.6 t in 2010 to 55.02 t in 2015, with a growth rate of 370%.

### HM emission flows through regional trade

From 2010 to 2015, the interprovincial embodied electricity flow caused by China's regional trade increased from 1216.9 TWh in 2010 to 1754.0 TWh in 2015, representing an increase of 44%. Meanwhile, the net HM emission flow caused by regional trade in China increased from 175.8 t (including 11.4 t Hg, 26.3 t As, 49.1 t Se, 55.2 t Pb, 1.1 t Cd and 32.7 t Cr) in 2010 to 227 t (including 13.8 t Hg, 38.6 t As, 60.1 t Se, 73.1 t Pb, 1.1 t Cd and 40.2 t Cr) in 2015.

**Figure 3:**
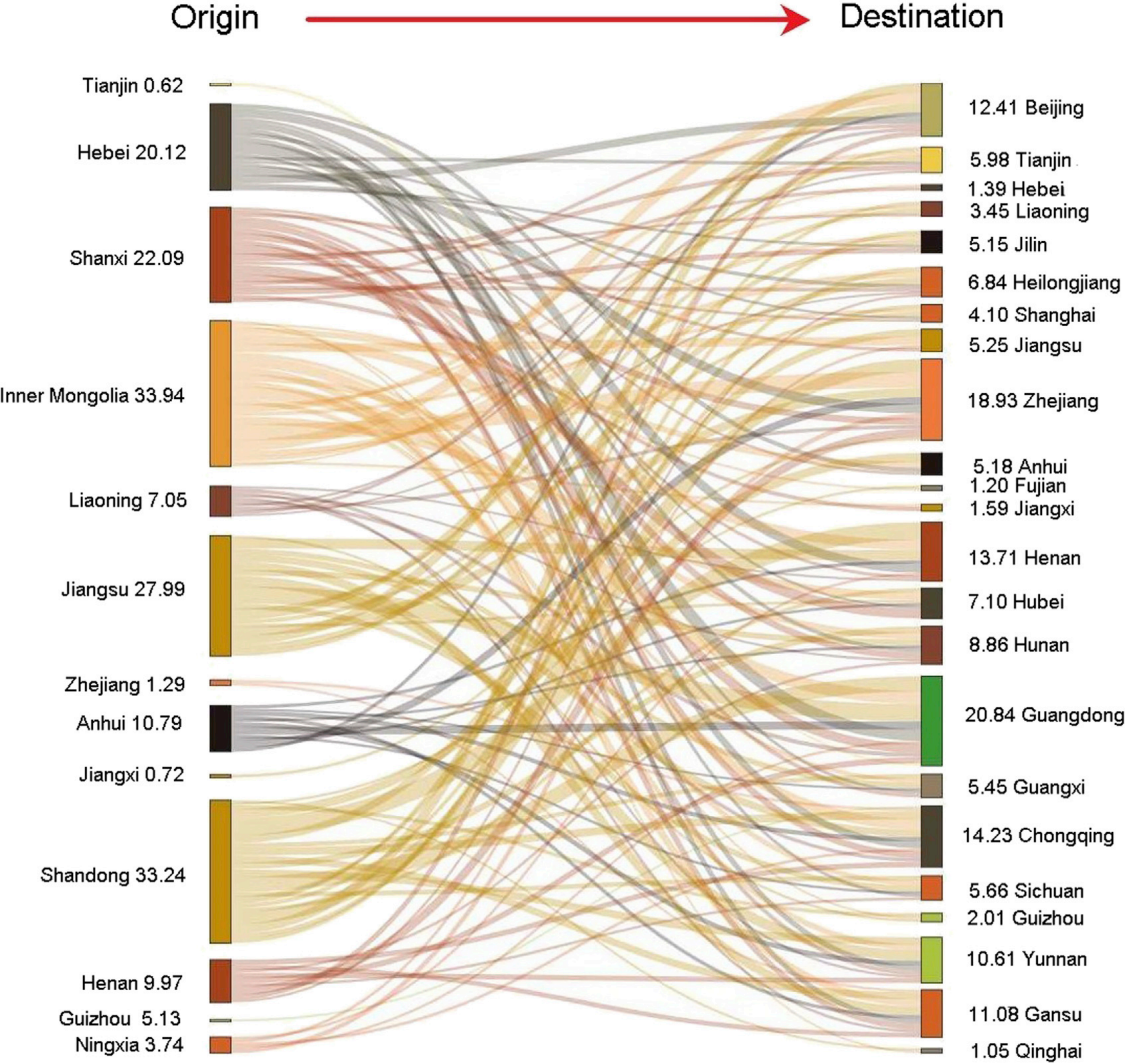
Net HM emission flows through regional trade in 2015 (Unit: t; those emission flows of less than 0.5 t are not shown; those emission flows in 2010 and 2012 are illustrated in Figures S3 and S4).

According to our results, the provinces with net inflows of HM emissions through regional trade are mainly economically developed, including Guangdong, Zhejiang, Shanghai, Beijing, and Chongqing, while the provinces with net outflows are mainly those with large-scale coal-fired power generations, such as Inner Mongolia, Shandong, Shanxi, Hebei, and Jiangsu (see Figure 3).

China's HM emission embodied in export gradually declined from 360 t (including 21.8 t Hg, 55.5 t As, 90.1 t Se, 109.6 t Pb, 1.9 t Cd and 80.7 t Cr) in 2010 to 257 t (including 15.4 t Hg, 39.8 t As, 66.9 t Se, 81.9 t Pb, 1.4 t Cd and 51.9 t Cr) in 2015. Jiangsu, Zhejiang, Guangdong, and Shandong and other eastern coastal provinces with more export manufacturers generated large-scale exported HM emissions.

### HM emissions under different perspectives vary substantially

**Figure 4:**
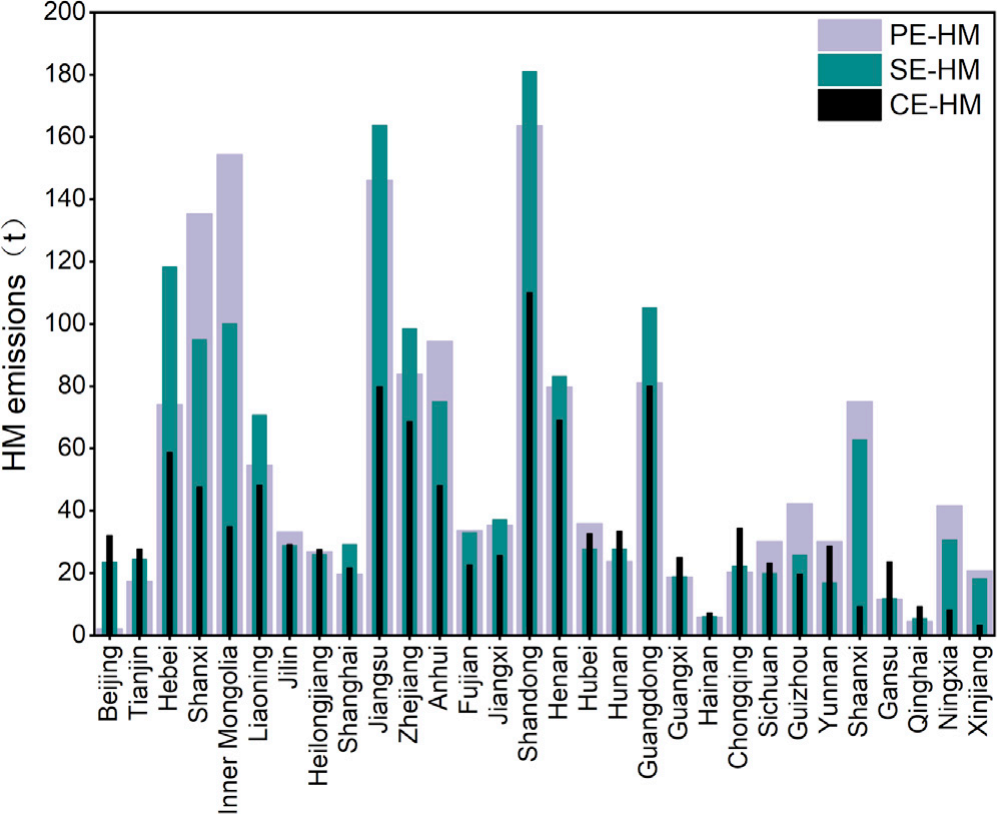
China's provincial HM emissions under different perspectives in 2015 (Provincial HM emissions in 2010 and 2012 are illustrated in Figures S5 and S6).

As shown in Figure 4, the distributions of PE-HM emissions in China are extremely uneven (Wei et al., 2021). Provinces with rich coal resources or large-scale coal-fired power generations (such as Inner Mongolia, Shanxi Shandong, Jiangsu, Guangdong, Henan, and Hebei) have higher PE-HM emissions. The total PE-HM emissions in the central and eastern regions are much higher than those in the western regions.

The SE-HM emissions of Beijing and Hebei were far higher than their PE-HM emissions as these two regions rely heavily on coal-fired electricity from other provinces, while the PE-HM emissions of Shanxi and Inner Mongolia were higher than their SE-HM emissions as these two provinces sold large volumes of coal-fired electricity to other regions. Such comparison results show that one region can transfer its environmental pressures to other power supply regions through electricity trade.

The provincial CE-HM emissions can be divided into two components: HM emissions induced by local final demand (including consumption and investment) and HM emissions induced by daily local household electricity consumption. Provinces with more developed economies and larger populations (such as Jiangsu, Zhejiang, and Guangdong) tend to release more CE-HM emissions. It is worth noting that some HM emissions are exported to foreign countries, so the total CE-HM emission is lower than the SE-HM and the PE-HM emissions. According to our results, per capita HM emissions (including productive and nonproductive activities) decreased significantly after undergoing slight increases in 2012. In 2010, China's per capita emissions of CE-Hg, CE-As, CE-Se, CE-Pb, CE-Cd, and CE-Cr were 6.4 × 10^−2^ g/person, 1.7 × 10^−1^ g/person, 2.6 × 10^−1^ g/person, 3.2 × 10^−1^ g/person, 5.3 × 10^−3^ g/person, and 2.3 × 10^−1^ g/person, while the per capita emissions of these 6 HMs decreased by 28.8%, 27.1%, 25.0%, 25.9%, 26.5%, and 30.9% (based on data for 2012) in 2015, respectively.

## Discussions

HM emissions cause more environmental damages and health losses in regions near the CFPPs due to their intensive pollutants deposition (Li et al., 2020). Provinces (such as Shanxi, Inner Mongolia, Anhui, Guizhou, Shaanxi, and Ningxia) suffer additional environmental costs and health losses for providing electricity to other regions. However, the current electricity price does not consider such environmental costs and health losses caused by power generation, and the prices of electricity from different sources are almost the same, which means that ecological and health losses in power supply areas cannot not compensated. In the future, environmental costs and health losses caused by power generation should be included in the electricity prices so that such environmental externalities can be internalized.

Our accounting results of HM emissions under three perspectives provide valuable policy implications for reducing HM emissions.

Small thermal power units are normally characterized by lower energy efficiency and high levels of various emissions (Cui et al., 2021). A large number of small thermal power units are still being operated in China (Li et al., 2020). They cannot meet the increasingly stringent environmental requirements even after ultralow emission transformation (Ministry of Ecological Environment, 2011). The cost of installing air pollution control devices (APCDs) is beyond the capacity of these small units. Therefore, provinces with more CFPPs (such as Shandong, Hebei, and Liaoning) should continue to promote the decommissioning of small thermal power units. In particular, these small thermal power units should be gradually phased out so that serious power shortages can be avoided. For example, in Shandong, the capacity of small units still accounts for more than 35% of the total capacity. If these small units were decommissioned over a short period, this would inevitably lead to a significant power shortage (China Electricity Council, 2011-2016a).

The HM emission factor of coal is one of the most important factors influencing HM emissions. To reduce the HM emission factors, measures including installing APCDs and setting coal quality standards for CFPPs should be taken, especially in provinces with high HM concentrations from coal (such as Yunnan and Guizhou). Power structure and generation efficiency also contribute to PE-HM emissions. Provinces with high renewable energy potential (such as Inner Mongolia) should continue to promote renewable power investment. At the 75th Session of the United Nations General Assembly, China's President Xi Jinping announced that China will achieve “carbon peak and carbon neutrality” status, whereby China will further accelerate the transformation of its power system. By 2030, the total installed capacity of wind and solar power will reach more than 1200 GW. This means that it is critical to establish concrete “carbon peak and carbon neutrality” goals for power sector by considering regional disparities.

Our results show that provinces with rich coal resources (such as Shanxi, Inner Mongolia, Anhui, and Guizhou) often have net electricity and HM emission outflows while more developed provinces tend to have net electricity and HM emission inflows. Therefore, cross-regional environmental supervision and governance should be initiated to coordinate regional development. Provinces with net inflows may consider providing financial and technological support to regions with net HM emissions outflows to reduce their PE-HM emissions, such as power plant construction technologies, energy audit and energy cascading technologies, professional training, and the installation and upgrading of APCDs.

Those eastern developed provinces, such as Shanghai, Jiangsu, Zhejiang, and Guangdong, have great advantages in their product supply chains and can obtain the embodied power supply through regional trade, leading to net HM emission inflows. These regions should promote green and low-carbon consumption behaviors and lifestyles, as well as circular economy (such as urban/industrial symbiosis, waste separation and recycling, eco-design, cleaner production, etc). In addition, since a large volume of CE-HM emissions in the central and western regions is induced by investment, it is necessary to control the scale of infrastructure construction and avoid irrational investment.

### Limitations of the study

Our study provides suggestions to allocate environmental responsibilities and achieve balanced regional development. There is a need to conduct further analysis on how much responsibilities should be taken by the inflow regions, and in what form to compensate those outflow regions. In addition, due to data limitations, our study only analyzed HM emissions from 2010 to 2015. To provide more timely policy recommendations, it is necessary to update the HM emission inventory in the future.

## Method details

### Regional PE-HM emissions and LMDI

We use the dynamic technology-based emission factor model (Guo et al., 2021; Zhang et al., 2015) to calculate the emission factors for Hg, As, Pb, Cr, Se, and Cd emissions from power generation.

(Equation 1) $$ef_{i,k}=HC_{i,k}\times \left(1-\sum_{l}P_{i,l}\times re_{l,k}\right)$$

where, *ef*_*i,k*_ is the emission factor in region *i* for *k* HM, *HC*_*i,k*_ is *k* HM content in one unit of coal consumption in region *i*, *P*_*i,l*_ is the proportion of *l* type of APCD combination in region *i*, and *re*_*l,k*_ is the removal efficiency of *l* type of APCD combination for *k* HM.

Then, the PE-HM emissions of the CFPPs in region *i* can be calculated as:

(Equation 2) $$e_{i,k}=fc_{i}\times ef_{i,k}$$

where, *e*_*i,k*_ is the amount of *k* HM emission from CFPPs in region *i*, and *fc*_*i*_ is the coal consumption of region *i*'s CFPPs.

Since data are only available for calculating HM emission factors for years 2010 and 2015, then a weight average method (Pan et al., 2011) is adopted to calculate the HM emission factors for year 2012.

(Equation 3) $$ef_{i,k}^{2012}=\frac{3}{5}ef_{i,k}^{2010}+\frac{2}{5}ef_{i,k}^{2015}$$

The LMDI approach is used in this study to quantify the contributions of various driving factors in PE-HM emissions.

(Equation 4) $$\begin{array}{l} e_{k}=\sum_{i}e_{i,k}=\sum_{i}ef_{i,k}\times fc_{i}=\sum_{i}ef_{i,k}\times \frac{fc_{i}}{CFP_{i}}\times \frac{CFP_{i}}{TP_{i}}\times TP_{i}=\sum_{i}ef_{i,k}\times ge_{i}\times es_{i}\times TP\\ ge_{i}=\frac{fc_{i}}{CFP_{i}},\;es_{i}=\frac{CFP_{i}}{TP_{i}} \end{array}$$

(Equation 5) $$\Delta e_{k}=e_{k}^{T}-e_{k}^{0}=\Delta e_{k}\left(ef\right)+\Delta e_{k}\left(ge\right)+\Delta e_{k}\left(es\right)+\Delta e_{k}\left(TP\right)$$

where *CFP*_*i*_ is the coal-fired power generation in region *i*, and TPi is the total power generation in region *i*. *Δe*_*k*_(*ef*)，*Δe*_*k*_(*ge*)，*Δe*_*k*_(*es*)，*Δe*_*k*_(*TP*) in Equation 5 represent the impact of HM emission factors of coal, coal-fired power generation efficiency, power generation structure, and total power generation, respectively.

According to the LMDI-I approach, contributions of these driving factors can be calculated as follows (Ang, 2005):

(Equation 6) $$\begin{array}{l} \Delta e_{k}\left(ef\right)=\sum_{i}L\left(e_{i,k}^{T},e_{i,k}^{0}\right)\cdot In\left(\frac{ef_{i}^{T}}{ef_{i}^{0}}\right)\\ \Delta e_{k}\left(ge\right)=\sum_{i}L\left(e_{i,k}^{T},e_{i,k}^{0}\right)\cdot In\left(\frac{ge_{i}^{T}}{ge_{i}^{0}}\right)\\ \Delta e_{k}\left(es\right)=\sum_{i}L\left(e_{i,k}^{T},e_{i,k}^{0}\right)\cdot In\left(\frac{es_{i}^{T}}{es_{i}^{0}}\right)\\ \Delta e_{k}\left(TP\right)=\sum_{i}L\left(e_{i,k}^{T},e_{i,k}^{0}\right)\cdot In\left(\frac{TP_{i}^{T}}{TP_{i}^{0}}\right) \end{array}$$

where *L*(*x*,*y*) = *x*−*y*/*lnx*−*lny* for *x*≠*y*, and *L*(*x*,*y*) = *x* for *x* = *y*.

### Regional SE-HM emissions

In this study, the network approach proposed by Qu et al. (2017) is adopted to calculate the provincial SE-HM emissions. This approach assumes that all the provinces are connected, and each province can produce, consume, buy and sell electricity. The sum of the electricity received by one province from any other provinces and the electricity locally produced is equal to the sum of the regional electricity outflow and consumption. In a model with *n* regions, the above relationship can be expressed as follows:

(Equation 7) $$x_{i}=p_{i}+\sum_{j=1}^{n}T_{j,i}=c_{i}+\sum_{j=1}^{n}T_{i,j}$$

where *x*_*i*_ is the total electricity flow of region *i*; *p*_*i*_ is the power generation in region *i*; *T*_*i,j*_ is the electricity generated in region *i* and consumed by region *j*; and *c*_*i*_ is the electricity consumption of region *i*. Then, we define the total power flow as a diagonal matrix as follows:

(Equation 8) $$\hat{x}=\left[\begin{array}{llll} x_{1} & 0 & \cdots & 0\\ 0 & x_{2} & \cdots & 0\\ \vdots & \vdots & \ddots & \vdots \\ 0 & 0 & \cdots & x_{n} \end{array}\right]$$

Based on the data of the provincial electricity flows in China, the electricity flow matrix *T* is obtained as follows:

(Equation 9) $$T=\left[\begin{array}{llll} 0 & T_{1,2} & \cdots & T_{1,n}\\ T_{2,1} & 0 & \cdots & T_{2,n}\\ \vdots & \vdots & \ddots & \vdots \\ T_{n,1} & T_{n,2} & \cdots & 0 \end{array}\right]$$

With the total electricity flow matrix $\hat{x}$ and electricity flow matrix *T*, we can obtain the direct outflow matrix *B* as:

(Equation 10) $$B={\hat{x}}^{-1}T=\left[\begin{array}{llll} 0 & \frac{T_{1,2}}{x_{1}} & \cdots & \frac{T_{1,n}}{x_{1}}\\ \frac{T_{2,1}}{x_{2}} & 0 & \cdots & \frac{T_{2,n}}{x_{2}}\\ \vdots & \vdots & \ddots & \vdots \\ \frac{T_{n,1}}{x_{n}} & \frac{T_{n,2}}{x_{n}} & \cdots & 0 \end{array}\right]$$

The element *B(i,j)* in the direct outflow matrix *B* is the proportion of the electricity flow from region *i* to region *j* to the total electricity flow of region *i*. According to Equation (7), we obtain the following:

(Equation 11) $$x=p+xB=\left[\begin{array}{llll} p_{1} & p_{2} & \cdots & p_{n} \end{array}\right]+\left[\begin{array}{llll} x_{1} & x_{2} & \cdots & x_{n} \end{array}\right]B$$

where *x* = [x_1_, x_2,_ … x_n_], and Equation (11) can be transformed into:

(Equation 12) $$x=p{\left[I-B\right]}^{-1}=pG$$

where I is an identity matrix, and *G* (*G = [I-B]-1 = I + B + B2 + B3 + …*) are the total interregional electricity flows (including the direct and indirect electricity flows). Its element *G(i, j)* is the proportion of electricity directly and indirectly flowing to region *j* to the power generated by region *i*.

Hence, we define the production-consumption matrix *H* as:

(Equation 13) $$H=G\hat{c}{\hat{x}}^{-1}$$

where is the diagonal matrix of the regional electricity consumption, and element (*i,j)* is region *i*'s electricity consumption; matrix *H* links the electricity generation and consumption in different regions, and element *Hi*, *j* is the proportion of the electricity generated in region *i* and consumed by region *j* to the total power consumption of region *j*.

The regional HM emissions from electricity generation can be linked with electricity consumption as follows:

(Equation 14) $$E^{HM}=E^{hm}\cdot H$$

where element *E*^*HM*^*(i,j)* are the HM emissions embodied in the electricity generated in region *i* and consumed in region *j*. Matrix *E*^*hm*^ is defined as follows:

(Equation 15) $$E^{hm}=\left[\begin{array}{llll} e_{1}^{hm} & 0 & \cdots & 0\\ 0 & e_{2}^{hm} & \cdots & 0\\ \vdots & \vdots & \ddots & \vdots \\ 0 & 0 & \cdots & e_{n}^{hm} \end{array}\right]$$

We can obtain the HM emissions embodied in region *i*'s electricity consumption (the SE-HM emissions) and obtain the SE-HM emission intensity of region *i*:

(Equation 16) $$E_{i}^{SE}=\sum_{j=1}^{n}E^{HM}\left(j,i\right)$$

(Equation 17) $$ef_{i}^{SE}=E_{i}^{SE}/c_{i}$$

where $E_{i}^{SE}$ are the SE-HM emissions of region *i* and $ef_{i}^{SE}$ is the HM emission intensity of the electricity consumed by region *i*.

### Regional CE-HM emissions

There are two sources of regional CE-HM emissions: the HM emissions embodied in the regional final demand (EFD) and those due to the residents' electricity consumption for daily life (ERE).

For the MRIO model with *m* regions and *n* sectors, the embodied emission intensities of the electricity-related HM emissions can be calculated using Equation (18) (Luo et al., 2020):

(Equation 18) $$E^{\text{intensity}}=C\cdot {\left(Y-Z\right)}^{-1}$$

where *E*^*intensity*^(1×mn) are the embodied HM emission intensities of the *m×n* sectors; *C(1×mn)* are the HM emissions embodied in the sectoral electricity consumption, which can be calculated by multiplying the sectoral electricity consumption and corresponding HM emission intensity $ef_{i}^{SE}$ in Equation 17; *Y(mn×mn)* is the diagonalized matrix of the total output of the *m×n* sectors; and *Z(mn×mn)* is the intermediate flow matrix in the MRIO table.

The HM emissions embodied in region *i*'s final demand *d*_*i*_ can be calculated as:

(Equation 19) $$E_{i}^{FD}=E^{\text{intensity}}\cdot d_{i}$$

where ${E_{i}}^{FD}$ are the HM emissions embodied in the final demand of region *i*.

Furthermore, we can calculate the HM emission flows through regional trade. Taking regions *i* and *j* as examples, the interregional electricity-related HM emission flows through regional trade from regions *i* to *j (EF*_*i,j*_*)* can be calculated as:

(Equation 20) $$EF_{i,j}=E_{i}^{\mathrm{int}ensity}\cdot d_{j}=C_{i}\left(Y-Z\right)\cdot d_{j}$$

where *C*_*i*_ (1×mn) are the HM emissions embodied in the sectoral electricity consumption of region *i* with a value of 0 for the other region sectors.

The HM emissions induced by the electricity consumption of the residents of region *i* for daily life ($E_{i}^{RE}$) can be calculated as:

(Equation 21) $$E_{i}^{RE}=ef_{i}^{SE}\cdot c_{i}^{RE}$$

where $c_{i}^{RE}$ is the electricity consumption of the residents of region *i* required for their daily life.

## STAR★METHODS

### Key resources table

This study did not use or generate any reagents.

### Resource availability

#### Lead contact

Further information and requests for resources and data should be directed to and will be fulfilled by the Lead Contact, Yong Geng (ygeng@sjtu.edu.cn).

#### Materials availability

This study did not use or generate any reagents.

#### Data and code availability

China's MRIO tables for 2010, 2012 and 2015 used in this study are publicly available (Liu et al., 2014, 2018b; Zheng et al., 2020). The electricity transmission data are obtained from the Compilation of Electric Power Industry Statistics for 2010, 2012 and 2015 (China Electricity Council, 2011-2016b). The data on power generation and consumption in different province are obtained from the China Power Yearbooks of 2011, 2013 and 2016 (China Electricity Council, 2011-2016a). China's provincial coal consumption data for power generation are obtained from the China Energy Statistical Yearbooks of 2011, 2013, and 2016 (National Bureau of Statistics of China, 2011-2016). Due to the limitation of data availability, our study doesn't include Tibet, Taiwan, Hongkong, and Macau. This study did not generate any codes. The preliminary data are available on request from the corresponding authors.
