# Supplementary material for: The reallocation effect of China's provincial power transmission and trade on regional heavy metal emissions
Source: iScience. 2021 May 11;24(6):102529. doi: 10.1016/j.isci.2021.102529 (PMC8188485; doi:10.1016/j.isci.2021.102529)
Supplement: Document S1. Figures S1–S6 and tables S1–S3 [file mmc1.pdf]

iScience, Volume *24*

Supplemental information

The reallocation effect of China's provincial power transmission and trade on regional heavy metal emissions

Wendong Wei, Zengcheng Xin, Yong Geng, Jiashuo Li, Mingtao Yao, Yaqin Guo, and Pengfei Zhang

Supplementary tables

Table S1. China's provincial HM emissions from CFPPs in 2010 (t). Related to the STAR Methods.

Region	Hg	As	Se	Pb	Cd	Cr
Beijing	0.32	0.79	1.85	1.99	0.04	1.09
Tianjin	1.22	2.87	6.76	7.61	0.17	4.28
Hebei	4.93	13.00	12.73	27.65	0.21	13.41
Shanxi	5.82	15.09	36.33	39.09	0.95	23.25
Inner Mongolia	10.04	32.18	15.85	54.64	0.20	21.03
Liaoning	3.93	14.40	7.54	22.57	0.14	15.78
Jilin	3.28	13.47	9.41	17.08	0.08	9.70
Heilongjiang	3.21	14.83	7.13	29.85	0.14	16.94
Shanghai	3.03	6.17	8.46	11.20	0.08	5.84
Jiangsu	9.83	18.94	39.03	47.11	0.68	32.36
Zhejiang	6.15	18.27	34.98	47.47	0.91	35.32
Anhui	8.42	7.14	42.73	13.08	0.13	21.16
Fujian	1.37	6.81	8.36	8.58	0.14	8.12
Jiangxi	1.40	4.39	13.09	7.58	0.17	7.61
Shandong	8.23	21.46	30.90	36.43	0.49	18.66
Henan	6.21	9.74	41.73	28.12	0.73	25.69
Hubei	2.32	4.76	13.00	15.38	0.27	10.42
Hunan	1.45	9.88	11.34	12.11	0.27	11.12
Guangdong	4.78	17.98	29.25	32.89	0.80	25.11
Guangxi	1.08	6.00	3.98	5.15	0.07	12.72
Hainan	0.16	0.56	1.26	1.44	0.04	1.00
Chongqing	1.73	3.03	5.02	6.67	0.23	4.25
Sichuan	2.42	4.88	7.36	10.45	0.52	8.57
Guizhou	5.13	4.79	10.68	0.00	0.33	8.91
Yunnan	3.44	7.69	3.75	13.89	0.22	14.33
Shannxi	3.65	8.65	18.82	27.57	0.57	19.69
Gansu	2.01	3.90	1.08	3.17	0.03	6.80
Qinghai	0.44	0.78	0.70	1.20	0.02	2.35
Ningxia	2.08	4.21	11.94	6.58	0.39	3.71
Xinjiang	0.43	2.06	0.42	0.80	0.03	1.27
TOTAL	108.51	278.71	435.47	537.32	9.06	390.47

Table S2. China's provincial HM emissions from CFPPs in 2012 (t). Related to the STAR Methods.

egion	Hg	As	Se	Pb	Cd	Cr
Beijing	0.32	0.89	1.91	2.27	0.05	1.31
Tianjin	1.28	2.91	6.98	7.74	0.17	4.24
Hebei	4.97	14.34	13.71	30.13	0.23	15.15
Shanxi	6.47	18.60	43.32	47.57	1.16	29.34
Inner Mongolia	13.08	42.88	21.36	73.84	0.28	28.58
Liaoning	3.87	14.41	7.57	22.59	0.14	15.55
Jilin	3.39	13.12	9.65	16.92	0.08	9.42
Heilongjiang	2.84	12.03	6.06	24.54	0.11	13.33
Shanghai	2.72	5.34	7.48	10.00	0.07	4.84
Jiangsu	11.41	22.00	45.40	54.99	0.79	36.26
Zhejiang	5.79	16.29	31.29	42.84	0.81	29.60
Anhui	9.31	7.85	48.53	14.51	0.15	22.99
Fujian	1.67	8.22	10.02	10.53	0.17	9.57
Jiangxi	1.32	4.21	12.87	7.30	0.17	7.33
Shandong	8.34	23.75	32.70	41.01	0.56	21.34
Henan	6.00	9.26	41.16	26.82	0.70	24.17
Hubei	2.07	4.21	11.60	13.95	0.25	8.96
Hunan	1.28	8.19	9.91	10.21	0.23	8.88
Guangdong	4.89	17.98	29.57	33.05	0.79	24.21
Guangxi	1.20	6.63	4.39	5.74	0.08	13.40
Hainan	0.21	0.72	1.66	1.86	0.05	1.27
Chongqing	1.50	2.82	4.59	6.04	0.20	3.82
Sichuan	2.48	5.61	7.99	11.84	0.60	9.90
Guizhou	5.26	6.40	12.01	4.57	0.35	9.25
Yunnan	3.32	8.97	3.95	16.40	0.26	18.50
Shannxi	3.87	9.08	19.61	29.50	0.60	20.16
Gansu	2.23	4.12	1.18	3.34	0.03	6.75
Qinghai	0.50	0.86	0.78	1.32	0.03	2.48
Ningxia	3.17	6.63	18.42	10.54	0.62	5.80
Xinjiang	0.83	4.30	0.88	1.66	0.06	2.81
TOTAL	115.58	302.61	466.56	583.64	9.78	409.24

Table S3. China's provincial HM emissions from CFPPs in 2015 (t). Related to the STAR Methods.

Region	Hg	As	Se	Pb	Cd	Cr
Beijing	0.09	0.28	0.54	0.71	0.01	0.43
Tianjin	1.01	2.13	5.28	5.72	0.12	2.96
Hebei	4.14	13.71	12.68	28.35	0.22	14.96
Shanxi	5.27	17.48	39.02	43.99	1.09	28.34
Inner Mongolia	10.57	36.18	18.38	63.88	0.24	24.96
Liaoning	3.22	12.35	6.52	19.35	0.12	12.96
Jilin	2.36	7.84	6.63	10.62	0.05	5.59
Heilongjiang	1.69	5.31	3.18	11.42	0.05	5.13
Shanghai	1.85	3.36	4.94	6.73	0.05	2.75
Jiangsu	9.84	18.99	39.32	47.93	0.68	29.27
Zhejiang	4.39	10.99	21.26	29.70	0.54	16.92
Anhui	8.35	6.98	45.78	13.11	0.13	19.98
Fujian	1.44	6.90	8.28	9.12	0.15	7.65
Jiangxi	1.34	4.39	13.98	7.67	0.18	7.72
Shandong	9.46	30.68	39.70	54.19	0.74	28.77
Henan	4.46	6.62	31.84	19.30	0.50	16.88
Hubei	1.84	3.63	10.22	12.67	0.22	7.29
Hunan	0.87	4.90	6.70	6.36	0.14	4.81
Guangdong	3.79	13.25	22.33	24.65	0.58	16.43
Guangxi	0.75	4.09	2.71	3.60	0.05	7.55
Hainan	0.22	0.71	1.69	1.86	0.05	1.20
Chongqing	1.49	3.13	4.95	6.42	0.22	4.01
Sichuan	1.71	4.50	5.99	9.36	0.47	8.00
Guizhou	4.32	7.54	11.50	10.79	0.30	7.78
Yunnan	1.55	5.18	2.07	9.56	0.16	11.60
Shannxi	3.57	8.24	17.51	27.68	0.55	17.38
Gansu	1.68	2.80	0.87	2.27	0.02	3.91
Qinghai	0.40	0.67	0.60	1.01	0.02	1.72
Ningxia	2.80	6.18	16.59	10.11	0.59	5.34
Xinjiang	1.42	8.35	1.75	3.21	0.11	5.92
TOTAL	95.90	257.35	402.80	501.33	8.37	328.22

Supplementary figures

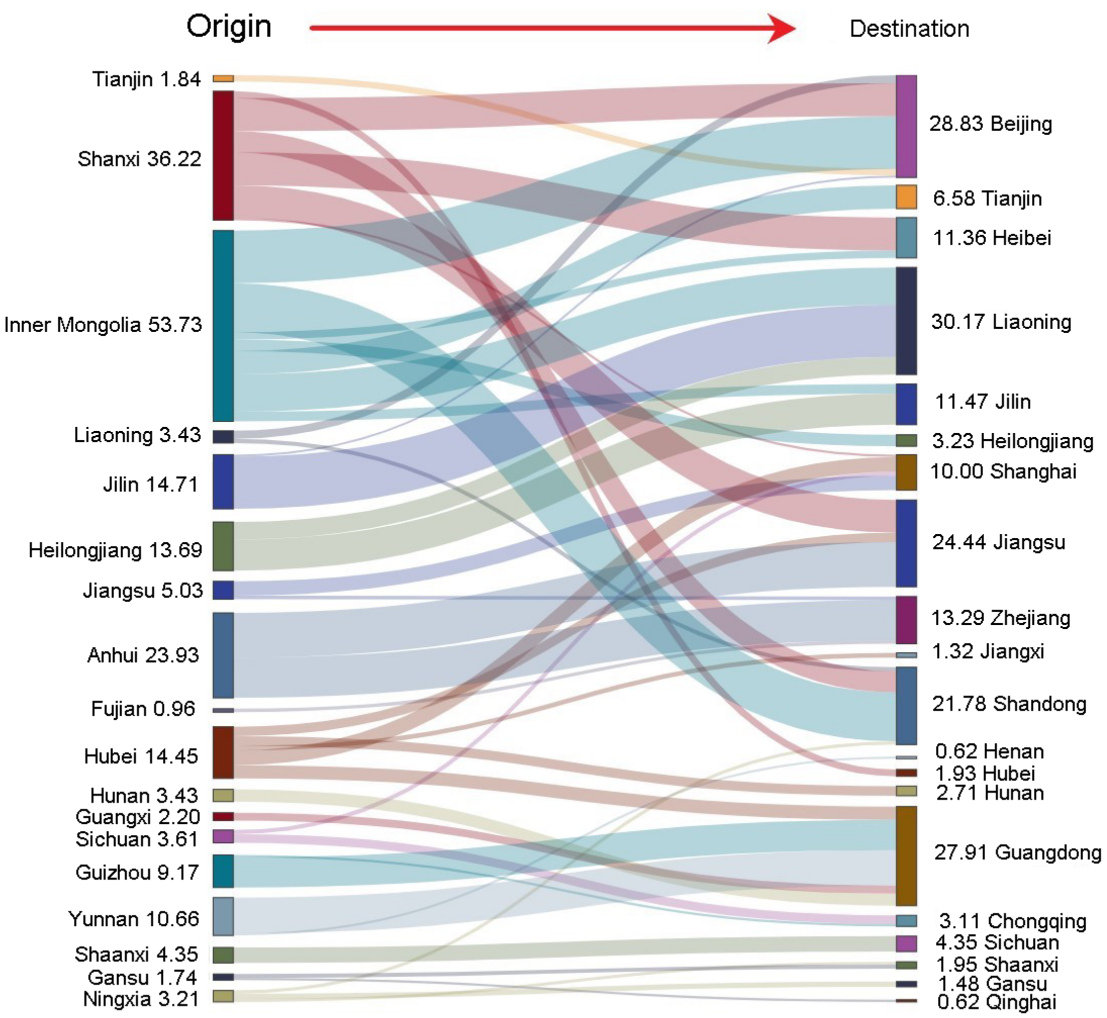

Figure S1. Net HM emission flows through interprovincial power transmission in 2010 (Unit: t; those emission flows of less than 0.1 t are not shown). Related to Figure 2.

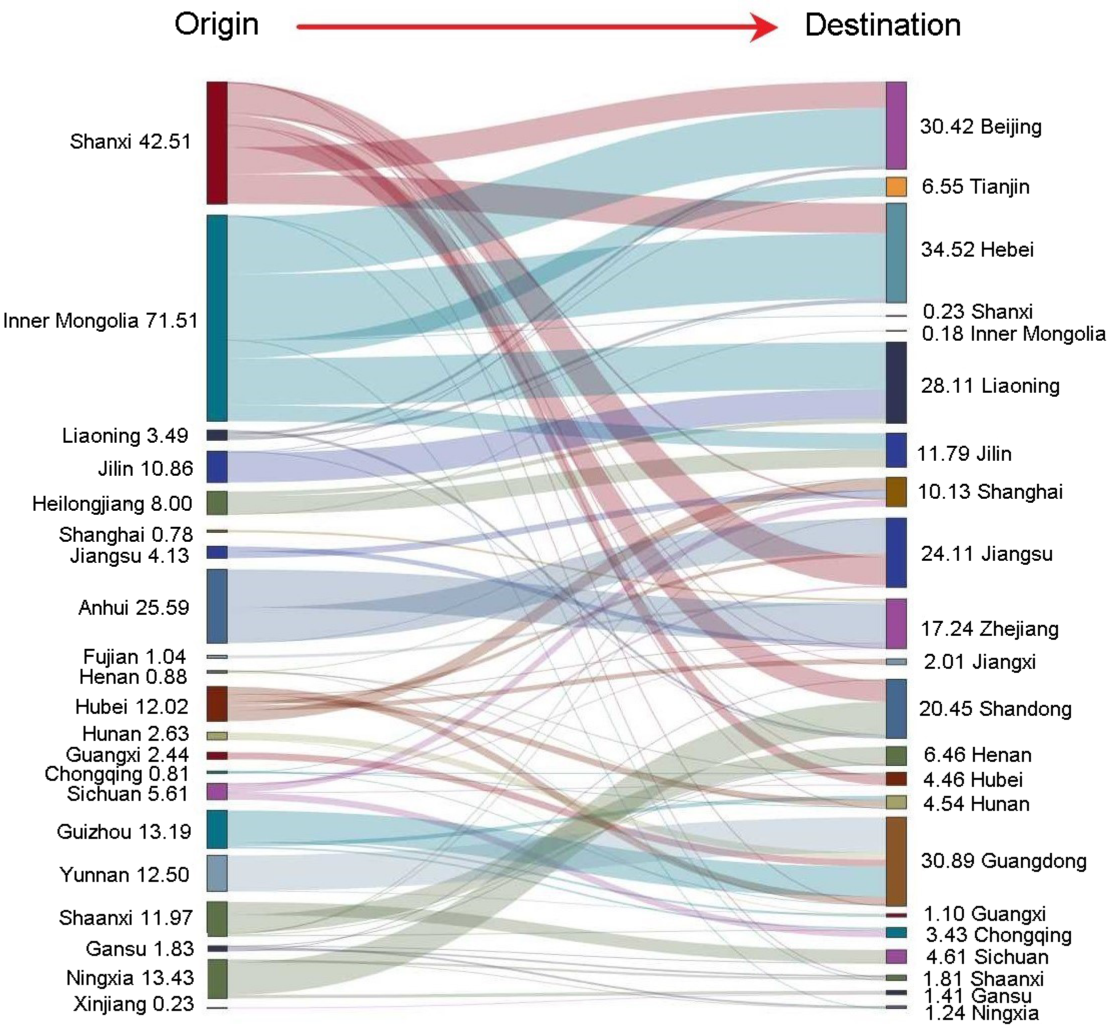

Figure S2. Net HM emission flows through interprovincial power transmission in 2012 (Unit: t; those emission flows of less than 0.1 t are not shown). Related to Figure 2.

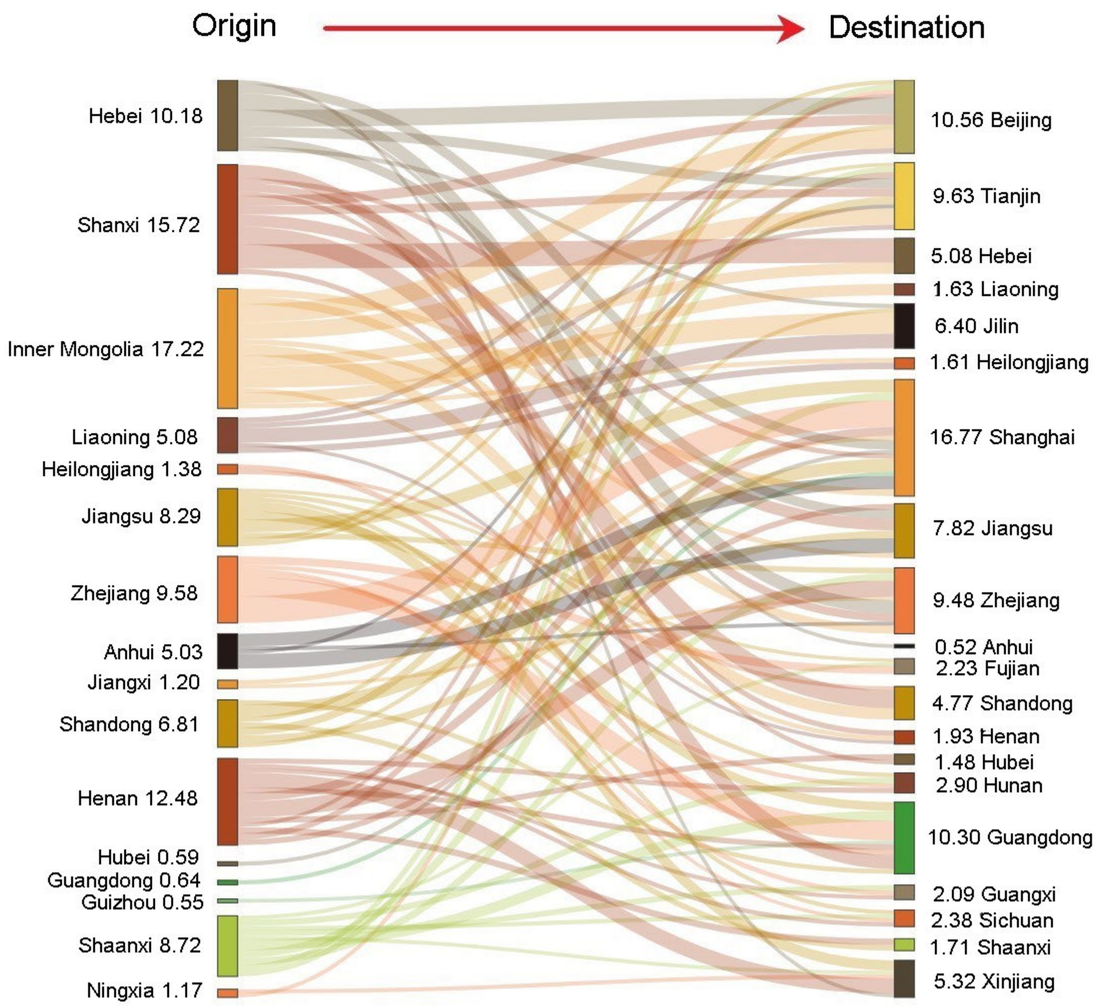

Figure S3. Net HM emission flows through regional trade in 2010 (Unit: t; those emission flows of less than 0.5 t are not shown). Related to Figure 3.

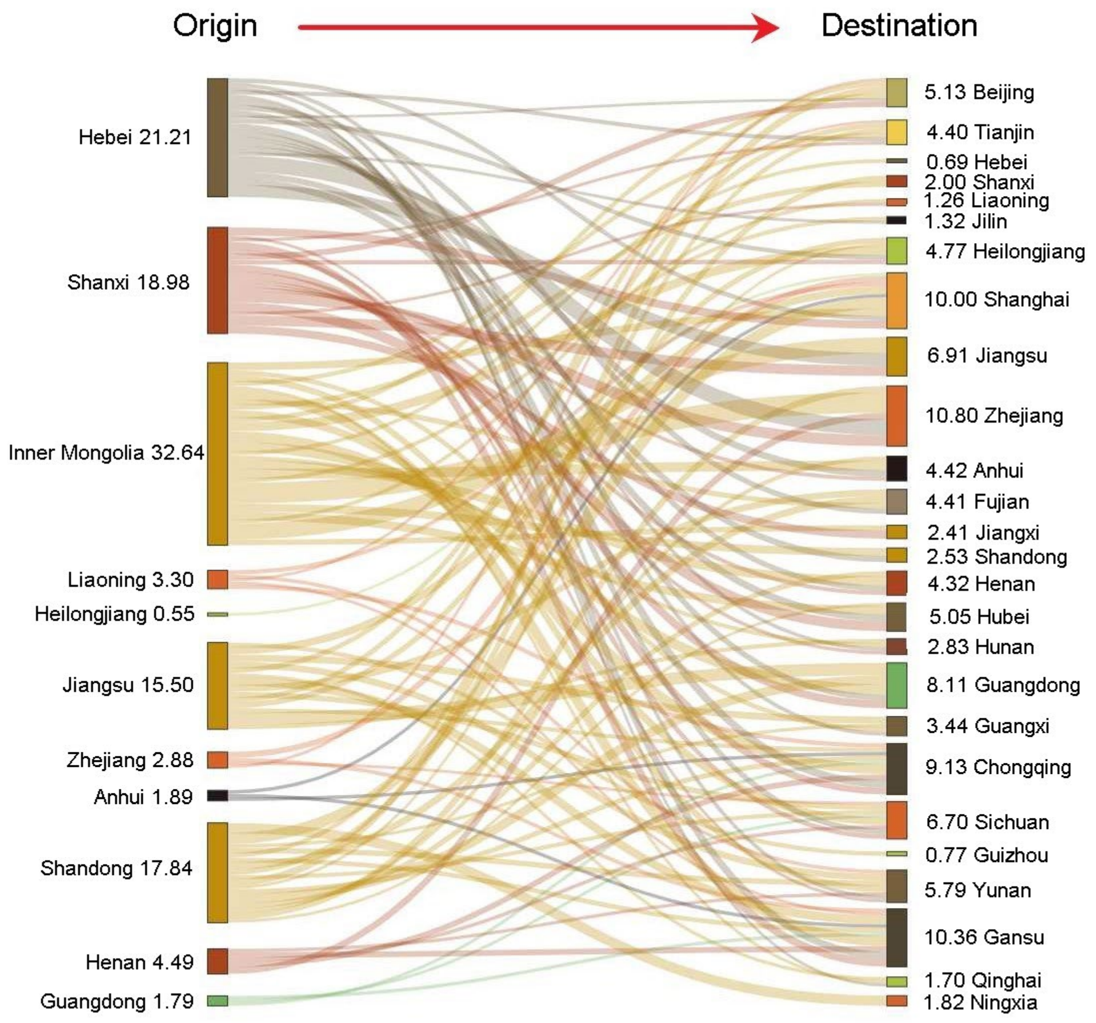

Figure S4. Net HM emission flows through regional trade in 2012 (Unit: t; those emission flows of less than 0.5 t are not shown). Related to Figure 3.

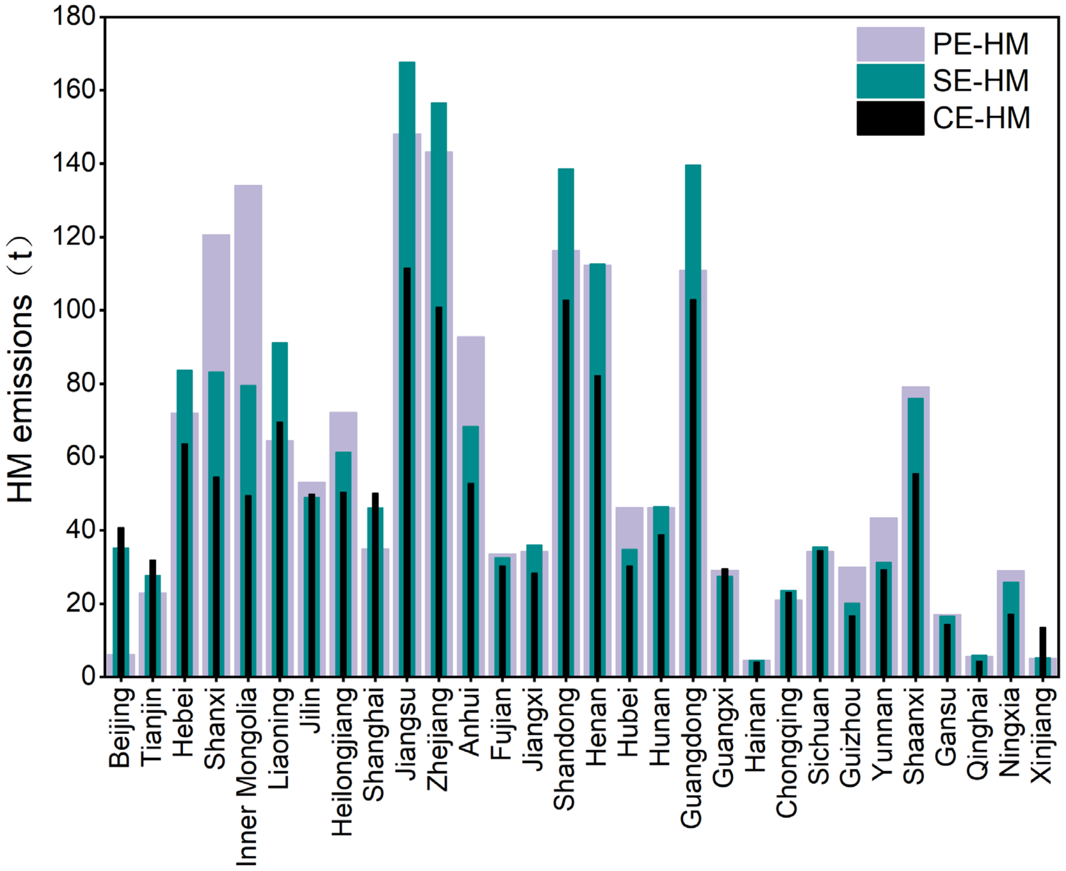

Figure S5. China's provincial HM emissions under different perspectives in 2010. Related to Figure 4.

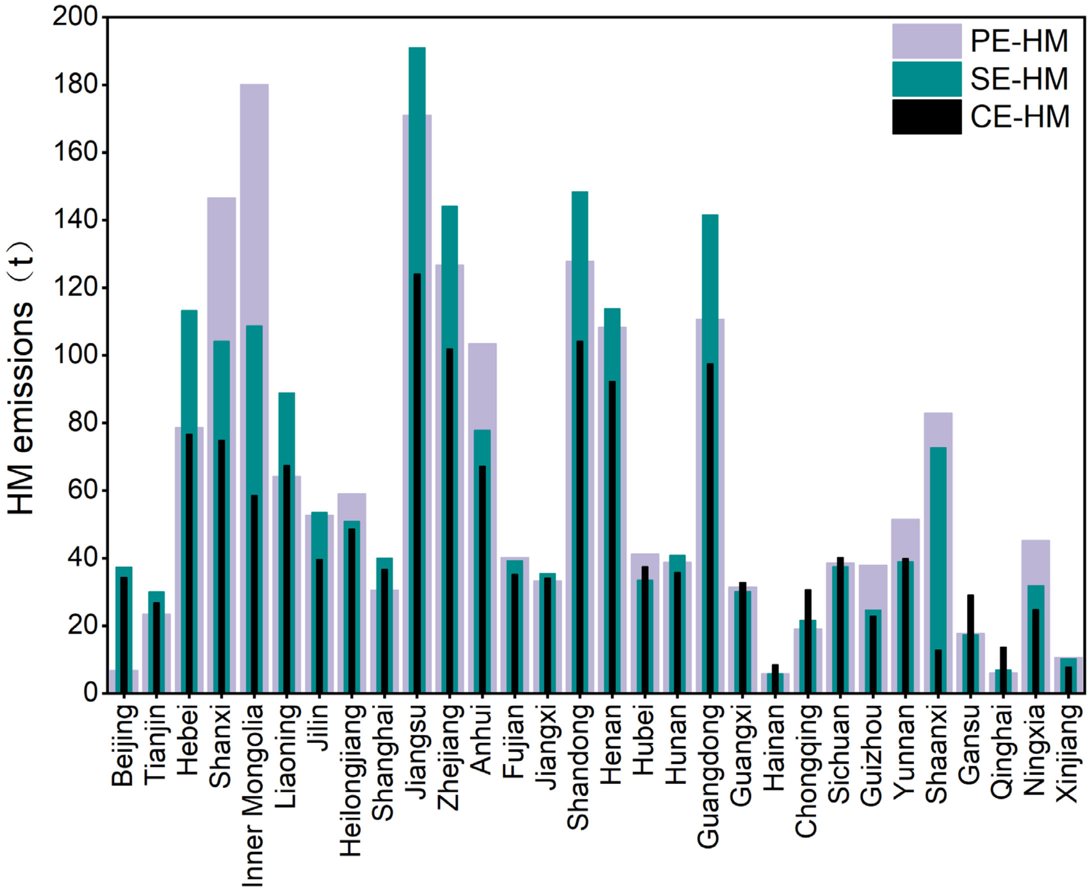

Figure S6. China's provincial HM emissions under different perspectives in 2012. Related to Figure 4.
